# Ovariectomy Induces Selective Alterations in Dura Mater Blood and Lymphatic Microvascular Network Architecture in Mice

**DOI:** 10.3390/cells14211647

**Published:** 2025-10-22

**Authors:** Olga V. Glinskii, Imad Eddine Toubal, Leike Xie, Sunilima Sinha, Kannappan Palaniappan, Vladislav V. Glinsky

**Affiliations:** 1 Department of Medical Pharmacology and Physiology, University of Missouri, Columbia, MO 65212, USA; glinskiio@health.missouri.edu; 2 Dalton Cardiovascular Research Center, University of Missouri, Columbia, MO 65211, USA; xiel@health.missouri.edu; 3 Harry S. Truman Memorial Veterans’ Hospital, Columbia, MO 65201, USA; 4 Department of Electrical Engineering and Computer Science, University of Missouri, Columbia, MO 65211, USA; itoubal@missouri.edu (I.E.T.); pal@missouri.edu (K.P.); 5 Department of Pathology and Anatomical Sciences, University of Missouri, Columbia, MO 65212, USA; sunilimasinha@health.missouri.edu

**Keywords:** ovariectomy, dura mater, microvasculature, lymphatics

## Abstract

Ovarian hormones are essential regulators of vascular homeostasis, yet their deficiency’s effects on the meningeal microvasculature remain incompletely understood. We used high-resolution imaging to assess the cranial dura mater (CDM) blood and lymphatic microvasculature in ovariectomized (OVX) and control (intact or sham-operated) mice, followed by morphometric analysis of microvessel architecture. Immunofluorescent staining and Western blotting were employed to evaluate markers of vascular remodeling and profibrotic signaling. Blood microvascular quantification revealed a significant reduction in total microvessel length two weeks post-OVX, primarily due to arteriolar, but not venular, shortening. At the same time, the lengths of individual segments of both arterioles and venules were also significantly decreased, indicating microvascular fragmentation. Despite these changes, total vessel surface area remained preserved, suggesting compensatory dilation, particularly in arterioles. OVX also increased overall vessel tortuosity, again selectively affecting arterioles. Region-specific analysis of lymphatic networks associated with the coronal suture (CS) showed significantly increased surface area of podoplanin-positive lymphatic vessels. Elevated α-smooth muscle actin (α-SMA) expression in vascular and stromal compartments in OVX animals, along with increased transforming growth factor beta (TGF-β) levels, indicated early profibrotic changes. These findings highlight the selective vulnerability of arterial and lymphatic microvascular structures to hormonal deficiency post-OVX and suggest an association between hormonal status, microvascular remodeling, and profibrotic alterations in the CDM.

## 1. Introduction

The cessation of ovarian hormone production during menopause or following ovariectomy induces widespread physiological changes, including vascular dysfunction and an increased risk of neurological disorders such as stroke and cognitive decline [1,2]. In rodents, OVX is a well-established model for mimicking hormonal changes in menopause and has been instrumental in elucidating the role of sex hormones in vascular regulation [3]. Estrogens are key modulators of vascular homeostasis, exerting their effects through endothelial cells, vascular smooth muscle cells (VSMCs), and the extracellular matrix (ECM) to regulate vascular tone, remodeling, and integrity [4,5,6]. At the cellular level, estrogens modulate VSMC proliferation, migration, and phenotypic switching—processes central to vascular repair and remodeling [7,8,9,10]. Estrogens also influence ECM composition by regulating protease activity and matrix protein expression, contributing to vessel stabilization [11,12]. Despite context-dependent variations in vascular outcomes, ovarian hormones are generally associated with protective effects on the vascular system.

Although estrogen’s role in systemic vascular regulation is well characterized, its influence on specialized vascular compartments, particularly the microvasculature of the meninges, remains less well understood. The cranial dura mater contains a dense and complex vascular network, including both blood and lymphatic vessels, essential for cerebrospinal fluid drainage, immune surveillance, neurovascular communication and CNS homeostasis. These vascular functions are highly dynamic, driven by continuous angioadaptation, and are sensitive to hormonal regulation [5,6,13]. Prior work from our group demonstrated that estrogen stabilizes the meningeal vasculature in large animal models by reducing permeability and pathological vessel morphology [14,15,16]. However, detailed insights into how the loss of ovarian hormones affects microvascular structure within the dura mater, particularly the cranial dura, are lacking.

Recent advances have identified the dura mater as a critical neurovascular and neuroimmune interface, containing both blood and lymphatic vessels that mediate bidirectional communication between the CNS and peripheral immune system [17,18,19]. Given the central role of the dura in CNS homeostasis, understanding how ovarian hormone deprivation influences meningeal microvascular architecture is of growing importance.

In this study, we used a mouse model of OVX-induced sex hormone deprivation to examine changes in the microvascular networks of the CDM. Using compartment-specific imaging and quantitative analysis, we evaluated blood and lymphatic vessel density, tortuosity, and associated tissue remodeling. These findings provide new insights into ovarian hormone-mediated regulation of meningeal vasculature and its potential implications for neurovascular and neuroimmune dysfunction in estrogen-deficient states.

Importantly, this work highlights the cranial dura as a hormonally sensitive vascular compartment and suggests that therapeutic strategies aimed at mitigating postmenopausal or age-related changes in estrogen signaling may help preserve microvascular stability. These insights could inform the development of targeted interventions to maintain neurovascular integrity and reduce CNS vulnerability in aging female populations.

## 2. Materials and Methods

### 2.1. Animals

All experimental procedures involving animals were approved by the University of Missouri Institutional Animal Care and Use Committee. Female C57BL/6J mice (2–4 months old) were obtained from Jackson Laboratory (Bar Harbor, ME, USA). At 2 months of age, mice underwent bilateral ovariectomy or sham surgery performed by Jackson Laboratory. Depending on the experiments (see below), either intact females (IF) or sham-operated animals (or both) were used as controls. No significant differences in any of the investigated parameters were observed between sham-operated and IF animals. Experimental analysis was conducted 2–3 weeks post-surgery.

Animals were allocated into two groups: (1) Control, sham-operated or IF mice with preserved ovaries, and (2) OVX mice with surgically removed ovaries. For random image analysis, 43 images were acquired from 4 IF mice and 50 images from 5 OVX mice.

An independent cohort of 3 sham-operated (30 images) and 3 OVX mice (30 images) was used for the analysis of lymphatic microvascular networks associated with the coronal suture area, previously discovered by our group [20]. A separate group of 4 sham-operated and 4 OVX mice was used for Western blot analysis.

### 2.2. Tissue Preparation and Image Acquisition

For imaging blood microvasculature, mice were perfused via the heart immediately after sacrifice with 1 mL of Krebs/BSA solution containing 20 μg/mL Alexa Fluor 594-conjugated soybean agglutinin (SBA) or wheat germ agglutinin (WGA) (Thermo Fisher Scientific, Waltham, MA, USA; Cat. No. W11261) to label blood vessels, as previously described [21]. For random imaging, the cranial skull with the attached dura mater was removed, mounted unfixed on slides, and imaged immediately.

For immunofluorescence experiments, dorsal cranium samples were fixed in 4% paraformaldehyde for 24 h, washed with PBS, and permeabilized overnight with 0.5% Triton X-100. Non-specific binding was blocked using 3% BSA in PBS, followed by immunostaining.

Lymphatic vessels were visualized using Syrian hamster anti-podoplanin (PDPL) antibody (Abcam, Boston, MA, USA; Cat. No. ab11936; 10 μg/mL, overnight at 4 °C), followed by Alexa Fluor 647-conjugated goat anti-Syrian hamster IgG (Abcam; Cat. No. ab180117; 5 μg/mL, 2 h at room temperature). Blood vessels were identified using rabbit anti-VEGFR3 antibody (NSJ Bioreagents, San Diego, CA, USA; Cat. No. R32892; 1:100), followed by Alexa Fluor-conjugated goat anti-rabbit IgG (Thermo Fisher Scientific; Cat. No. A11072). Vascular smooth muscle cells were labeled with FITC-conjugated anti-α-smooth muscle actin (α-SMA) antibody (Sigma-Aldrich, St. Louis, MO, USA; Cat. No. F3777-.2 ML; 3 μg/mL).

Samples were washed three times with PBS (5 min each) and mounted using Ibidi Mounting Medium for fluorescence microscopy (Ibidi Inc., Fitchburg, WI, USA; Cat. No. 50001). Imaging was performed using a Fluoview FV1000 confocal microscope (Olympus, Tokyo, Japan) with FV10-ASW software version 2.1. Z-stacks (180–200 images, 512 × 512 pixels) were acquired using a 20× objective at 1 μm intervals.

**Figure 1:**
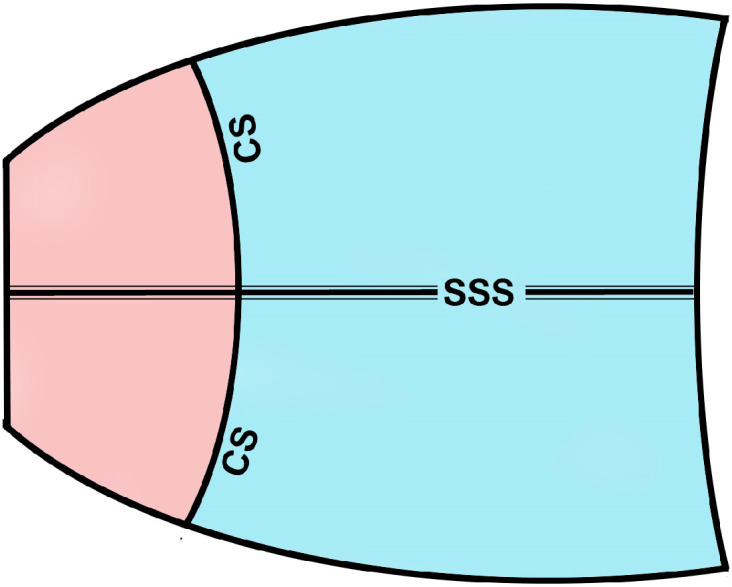
Schematic representation of the mouse skull cap with cranial dura mater. SSS, superior sagittal sinus; CS, coronal suture. The frontal area is indicated in pink, and the parietal area in blue.

To analyze the microvascular networks along the CS, where PDPL-positive lymphatic networks were reported [20], 10 images were taken along the entire CS area in each animal (Figure 1).

### 2.3. Image Processing and Microvascular Analysis

**Figure 2:**
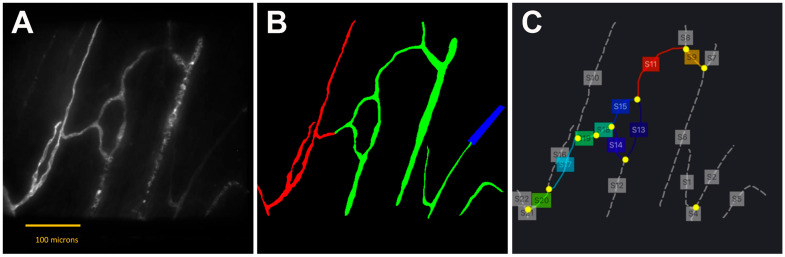
Segmented microvasculature image fused from a 3D confocal stack. (**A**) Original image. (**B**) Annotated image showing arteriole segments (red), venular segments (green), and undetermined regions (blue), which could not be annotated due to visual ambiguity. (**C**) Skeletonized image with vascular segments (gray dotted lines with labeled boxes) and branching points (yellow dots). Complete vessel segments are shown as colored solid lines with labeled boxes. Scale bar: 100 µm.

Both manual and semi-automated segmentation approaches were applied to identify blood vessels [21,22,23]. Each image was annotated to generate a two-class mask, distinguishing arterioles (Figure 2B, red) and venules (Figure 2B, green), in addition to the background (black).

To analyze vascular architecture, a skeletonization algorithm was applied to extract vessel segments and nodes (Figure 2C; segments-grey dotted lines, nodes-yellow dots). Two node types were defined: (i) branching points, where a vessel splits into two or more branches; and (ii) transition points, representing anatomical transitions between arterioles and venules.

Complete vessel segments were defined as regions bounded by two branching or transition points (Figure 2C, solid lines and colored boxes). Quantitative metrics were computed exclusively for complete segments using a custom-developed automated microvascular analysis tool. Tortuosity was calculated as the ratio of the actual vessel path length to the straight-line distance between endpoints. Vessel area was calculated as the fractional area occupied by vessels within the total image area.

To evaluate vascular smooth muscle cell (VSMC) coverage of meningeal blood vessels, deep learning-based segmentation cascades were used to identify blood vessel boundaries, as previously described [20,22,23]. The blood vessel network area was measured in each image. α-SMA-associated immunofluorescence signal was then quantified over the segmented vessel area, and the ratio of α-SMA signal to vessel area was calculated to determine VSMC coverage in blood vessels. To assess non-vessel-associated α-SMA expression, the difference between total α-SMA signal in the image and vessel-associated α-SMA signal was quantified.

### 2.4. Western Blot (WB) Analysis

Western blot analysis included samples from 4 sham-operated and 4 OVX mice. Dura mater tissues attached to cranial bones were lysed using Lysis buffer (RayBiotech, Peachtree Corners, GA, USA; Cat. No. AA-KYS-10 mL) with protein inhibitor cocktail (RayBiotech; Cat. No. AA-PI). Protein concentrations were determined using Pierce^TM^ BCA Protein Assay kit (Thermo Fisher Scientific; Cat. No. 23227). Equal amounts of the protein from each sample (15 μg) were resolved on NuPAGE 4–12% Bis-Tris gels (Thermo Fisher Scientific; Cat. No. NP0321) and transferred to nitrocellulose membranes (Thermo Fisher Scientific; Cat. No. LC2001). The membranes were sequentially probed with primary antibodies directed against TGFβ1 (Santa Cruz Biotechnology, Dallas, TX, USA; Cat. No. sc-130348, 1:1000) in conjunction with secondary antibodies (LI-COR Inc., Lincoln, NE, USA; Cat. No. 925-32212) and detection using Odyssey DLX (Li-COR Inc) imaging system. Membranes were subsequently stripped and reprobed with anti-β-Actin antibody (Abcam, Cambridge, MA, USA; Cat. No. ab8227), which served as a loading control for normalization of target protein expression.

### 2.5. Statistical Analysis

For the statistical analysis, the data from each annotated image were averaged for each experimental animal. Next, the individual mean values for each animal were used in statistical analysis to determine the *p* value. Statistical analyses were performed using GraphPad Prism version 10.5.0 (GraphPad Software, La Jolla, CA, USA). An unpaired *t*-test was used to assess differences between experimental groups. A *p*-value < 0.05 was considered statistically significant.

## 3. Results

### 3.1. Ovariectomy Induces Selective Alterations in Microvascular Architecture

Our comprehensive quantitative assessment revealed that OVX selectively alters the microvascular architecture of the cranial dura mater, particularly within the arteriolar compartment of vascular networks. This effect is evident across following key vascular parameters, highlighting the sensitivity of arterial component of microvascular networks to ovarian hormone deficiency.

#### 3.1.1. Vessel Length

**Figure 3:**
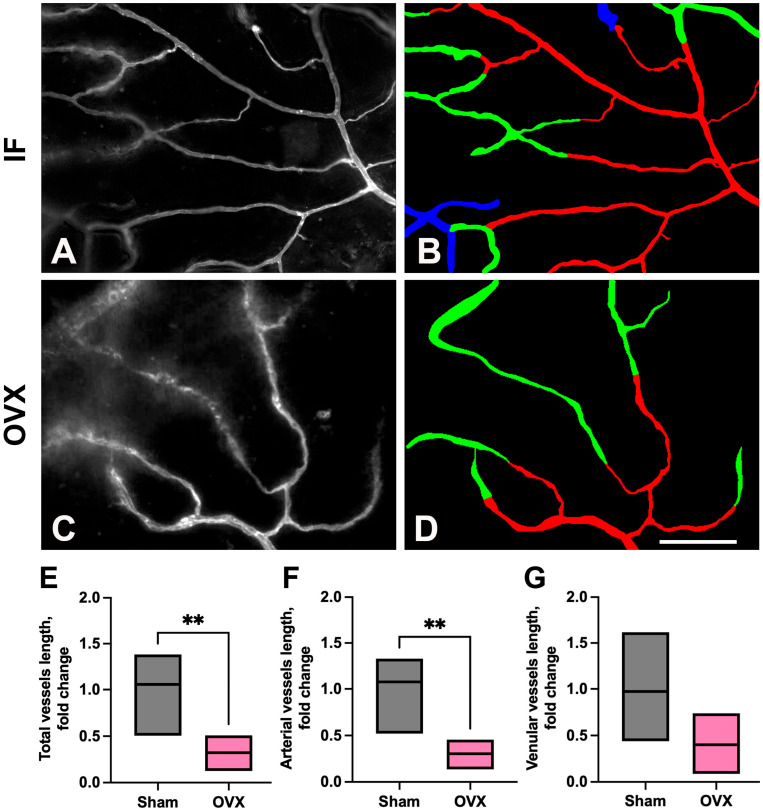
Meningeal vascular remodeling at 2 weeks post-ovariectomy. (**A**,**C**) Representative images from IF and OVX mice, respectively. (**B**,**D**) Corresponding annotated images showing arteriole segments (red), venular segments (green), and undetermined regions (blue), which could not be annotated due to visual ambiguity. (**E**) Total vessel length was significantly reduced in OVX mice (*n* = 5 animals) compared to IF (*n* = 4 animals) controls (*p* = 0.008). (**F**) Arteriolar vessel length was decreased in OVX mice (*p* = 0.004), whereas venular vessel length (**G**) remained unchanged, indicating a selective effect of ovarian hormone depletion on arteriolar vessels. Statistical significance was determined using an unpaired *t*-test. Scale bar in D: 100 µm. ** *p* < 0.01.

To quantify vessel length within microvascular networks, each original image (Figure 3A,C) was annotated to generate a two-class mask, distinguishing arterioles (Figure 3B,D, red) and venules (Figure 3B,D, green), with the black background excluded (see Methods: Image Processing and Microvascular Analysis). A significant reduction in total meningeal vessel length was observed in OVX mice two weeks post-surgery compared to IF controls (Figure 3E). This reduction was primarily attributable to a decrease in arteriolar vessel length (Figure 3F), whereas venular vessel length remained unchanged (Figure 3G). Thus, initial analyses demonstrated a significant reduction in total microvessel length post-OVX, driven predominantly by a decrease in arteriolar length (see Supplementary Materials Table S1: FILE_Blood vessel morphometry_IF_vs_OVX). This finding underscores a selective vulnerability of arteriolar structures to ovarian hormone deficiency.

#### 3.1.2. Vessel Segment Length

**Figure 4:**
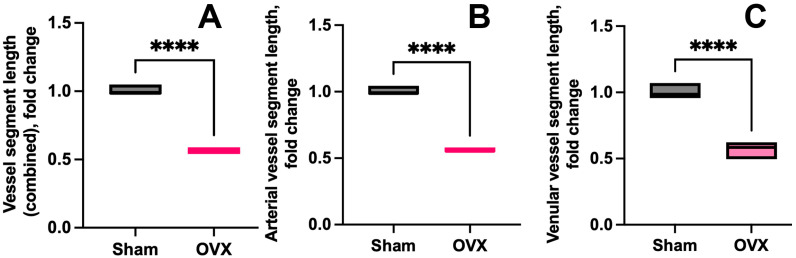
Vessel segment length alterations following OVX. (**A**) Overall vessel segment length was significantly reduced in the OVX mice (*n* = 5 animals) compared to IF controls (*n* = 4 animals), *p* < 0.0001. This reduction included both arteriolar (**B**) and venular (**C**) segment lengths, suggesting potential fragmentation or shortening within the meningeal microvascular network. Statistical significance was determined using an unpaired *t*-test. **** *p* < 0.0001.

To further support this observation, a skeletonization algorithm was applied to extract vessel segments and nodes (see Methods: Image Processing and Microvascular Analysis). Quantitative metrics were computed exclusively for complete segments, defined as regions bounded by two branching or transition points [Methods, Image Processing and Microvascular Analysis]. Quantitative analysis of vessel morphology in OVX mice revealed significant reductions in dura mater vessel segment length (Figure 4A), including both arteriolar (Figure 4B) and venular components (Figure 4C), compared to IF controls (*p* = 0.0001 for all). These changes suggest fragmentation or shortening within the meningeal microvasculature (see Supplementary Materials Table S1: FILE_Blood vessel morphometry_IF_vs_OVX). At the same time, vessel surface area remained unchanged implying compensatory dilation or structural reorganization to preserve perfusion, particularly within the arterial part of microvascular networks.

#### 3.1.3. Vessel Tortuosity

**Figure 5:**
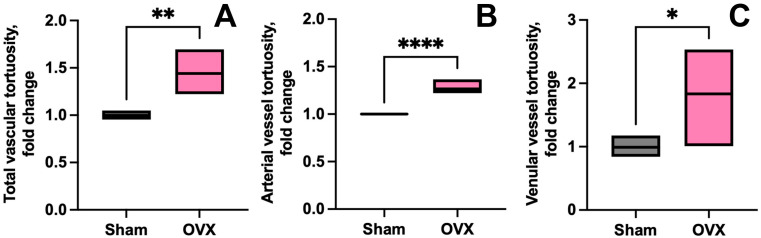
Ovariectomy-driven alterations in meningeal vascular tortuosity. (**A**) Overall meningeal tortuosity is significantly increased in OVX mice compared to intact female (IF) controls (*p* = 0.002). (**B**) This increase is primarily driven by higher tortuosity in arterial segments (*p* = 0.0001). (**C**) A smaller, but significant increase is also observed in venular segments (*p* = 0.04). IF mice (*n* = 4 animals), OVX mice (*n* = 5 animals). Statistical analysis was performed using an unpaired *t*-test. **** *p* < 0.0001, ** *p* < 0.01, * *p* < 0.05.

In line with these compensatory responses, we observed an increase in vessel tortuosity following OVX. Post-OVX animals exhibited a significant increase in overall meningeal vessel tortuosity across all compartments of the microvascular network compared to IF controls (Figure 5A, *p* = 0.002). However, this increase was primarily driven by markedly greater tortuosity in the arterial segments of the microvascular network (Figure 5B, *p* < 0.0001) than in the venular segments (Figure 5C, *p* = 0.04), indicating that arterial components are more sensitive to ovarian hormone deficiency (see Supplementary Materials Table S2: FILE_Vessel_tortuosity_IF_vs_OVX). These architectural adaptations may reflect a compensatory mechanism to preserve vascular function under hormone-deficient conditions, further underscoring the unique role of estrogens in maintaining vessel integrity.

### 3.2. Lymphatic Network Remodeling Within Coronal Suture-Associated Areas

Next, we investigated whether OVX-induced vascular changes extended to lymphatic vasculature. For this purpose, we specifically focused on the CS regions, where we previously identified PDPL-positive lymphatic networks [20]. Our data showed significant increase in the surface areas of PDPL-positive lymphatic vascular networks in the CS region.

**Figure 6:**
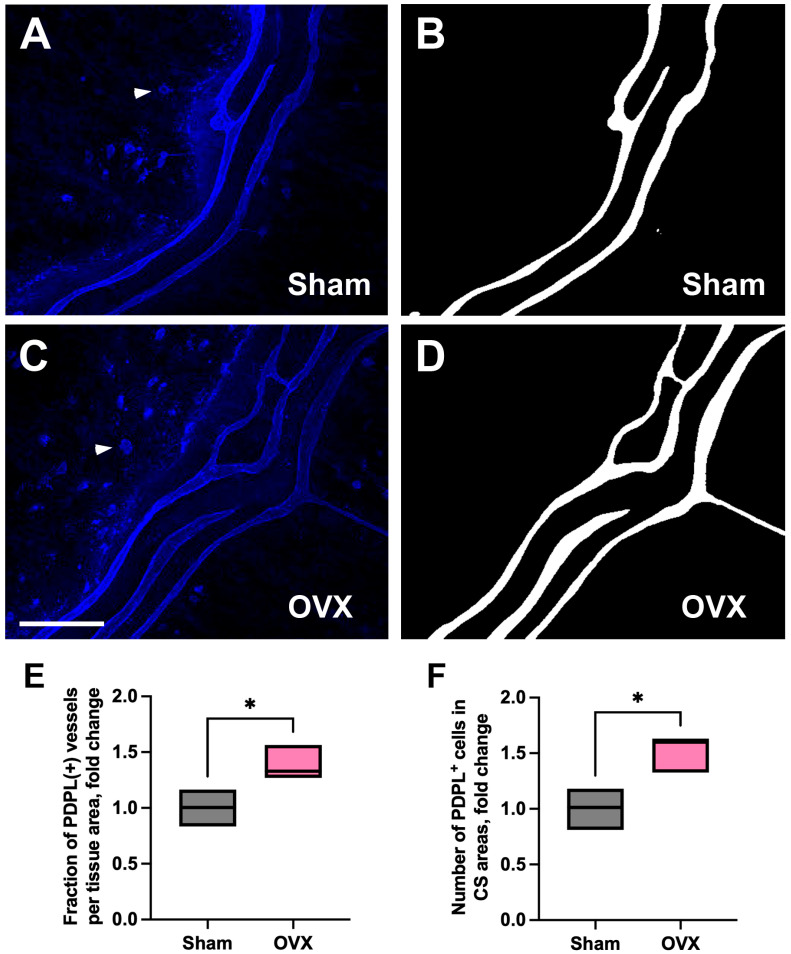
Region-specific remodeling of the lymphatic vasculature in the lateral zones of CS-associated dura following OVX. (**A**,**C**) Representative original images of PDPL-positive lymphatic vessels and cells in lateral CS regions from sham-operated and OVX mice. (**B**,**D**) Annotated vessels from original images. (**E**) Fraction of podoplanin (PDPL)-positive lymphatic vessels per image area was significantly increased in OVX mice compared to sham controls (*p* = 0.042). (**F**) Number of PDPL^+^ cells was significantly increased in OVX animals (*p* = 0.02). Statistical significance was assessed using an unpaired *t*-test. A *p*-value < 0.05 was considered statistically significant. * *p* < 0.05. Scale bar in C: 100 µm.

PDPL-positive lymphatic vessels from original images of the lateral CS regions of sham and OVX mice (Figure 6A,C) were annotated (Figure 6B,D) and quantitatively analyzed [21,22,23]. This analysis revealed that OVX-induced expansion of PDPL-positive lymphatic networks in CS-associated areas is characterized by a significantly increased fraction of PDPL-positive lymphatic vessels per image area compared to sham controls (Figure 6E) with no significant difference in mean segment length (see Supplementary Materials Table S3: FILE_OVX_vs_sham_Coronal_Suture_PDPL+ vessel_area & cell count).

In addition, OVX mice exhibited a significant increase in the number of podoplanin-positive cells (white arrowhead) within the CS regions compared to control animals (Figure 6A,C,F). These cells are thought to be lymphatic endothelial progenitors involved in lymphangiogenesis [24].

Collectively, these findings suggest a coordinated remodeling response of CS-associated lymphatic vasculature to ovarian hormone withdrawal, which is distinct from the alterations observed in the arterial blood networks.

### 3.3. Vascular and Tissue Stromal Alterations in α-SMA Expression

**Figure 7:**
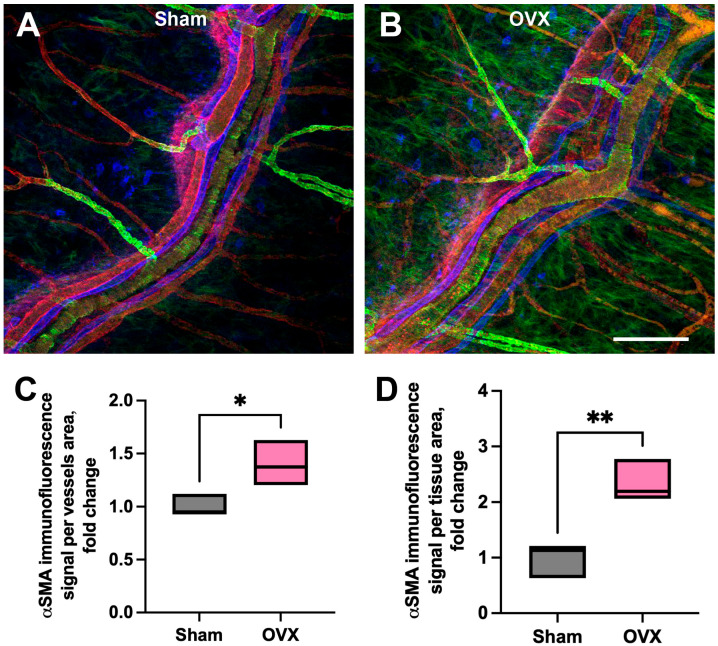
Increased α-SMA expression in CDM blood vessels and connective tissue stroma following OVX. (**A**,**B**) Representative immunofluorescence images of the CDM from sham-operated (**A**) and OVX (**B**) mice. VEGFR3-positive blood vessels are shown in red, α-SMA in green, and PDPL-positive lymphatic vessels in blue. (**C**,**D**) Quantitative analysis of α-SMA fluorescence intensity reveals a significant increase in α-SMA signal in blood vessels (**C**) and within CDM connective tissue stroma (**D**) in OVX mice compared to sham controls (*p* = 0.04 and *p* = 0.009). Statistical significance was determined using an unpaired *t*-test. * *p* < 0.05, ** *p* < 0.01. Scale bar in (**B**): 100 µm.

**Figure 8:**
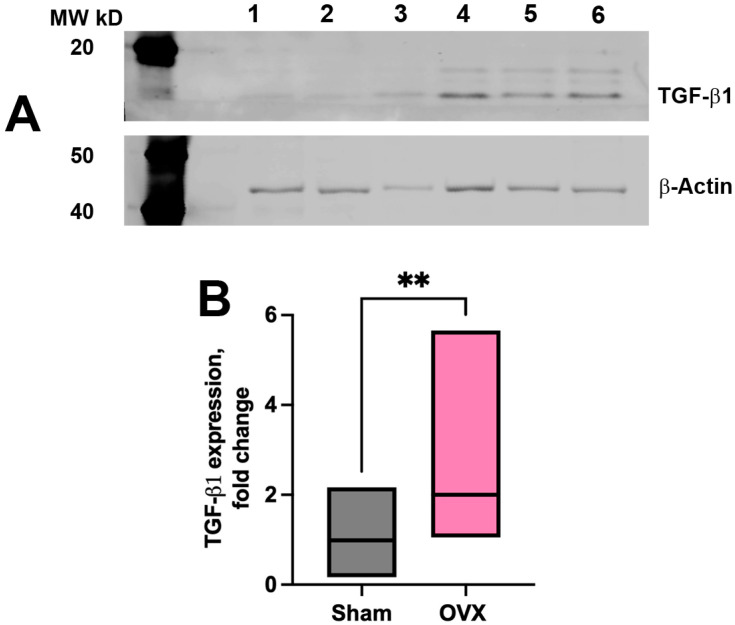
Western blot analysis of TGF-β1 protein expression in sham and OVX groups. (**A**) Representative Western blot of TGF-β1 protein in CDM tissue lysates. Samples containing 15 µg of total protein from sham (lanes 1–3) and OVX (lanes 4–6) groups were separated by SDS-PAGE and probed with anti-TGF-β1 antibody. The 17 kDa band corresponding to TGF-β1 was quantified by densitometry while β-actin was used as a loading control to normalize TGF-β1 expression. (**B**) Quantification of TGF-β1 expression levels normalized to β-actin. Data represent the mean ± SEM from four independent experiments. Statistical significance was determined using an unpaired *t*-test. *p* < 0.05 was considered statistically significant. ** *p* < 0.01.

Along with structural remodeling of blood and lymphatic microvasculature, we identified molecular changes in both the vascular wall and the surrounding stromal tissue (Figure 7). OVX mice exhibited enhanced α-SMA expression along blood vessels (Figure 7B,C) compared to sham-operated animals (Figure 7A,C), indicating upregulation of vascular smooth muscle components (see Supplementary Materials Table S4: FILE_aSMA coverage_OVX_vs_Sham), potentially in response to altered hemodynamics. In addition to vascular alterations, pronounced remodeling was observed within the connective tissue of the cranial dura following OVX. These changes were characterized by increased α-SMA immunoreactivity within the CDM stromal compartment in OVX mice relative to sham controls (Figure 7B,D). Furthermore, Western blot analysis revealed significantly elevated TGF-β1 expression in the OVX group (Figure 8A, lanes 4–6) compared to controls (Figure 8A, lanes 1–3). Cumulative data from four independent Western blot experiments confirmed a significant increase in TGF- β1 expression in OVX group (see Supplementary Materials Table S5: FILE_TGF-beta densitometry_OVX_vs_sham) compared to the sham controls (Figure 8B). The concurrent enhancement of α-SMA immunoreactivity within the dural stroma and elevated TGF-β1 levels suggest a shift toward a profibrotic tissue environment 2–3 weeks post-ovariectomy.

## 4. Discussion

Our findings demonstrate that OVX leads to significant, compartment-specific remodeling of the CDM blood microvasculature, with particularly pronounced alterations in the arteriolar networks. This observation aligns with the well-established role of sex hormones, particularly estrogens, in regulating cerebrovascular function and integrity. Previous studies have shown that cessation of ovarian hormone production, whether gradual as in menopause, or abrupt, following OVX, disrupts cerebral blood flow and increases the vulnerability of the brain vasculature to injury and disease, contributing to a heightened risk of neurological disorders such as stroke, migraine, and neurodegeneration [1,16,25,26]. The integrity and function of microcirculatory networks are essential for maintaining tissue homeostasis, as they govern the exchange of oxygen, nutrients, and metabolic waste between the bloodstream and surrounding tissues [27]. Notably, microvascular dysfunction has emerged as a key feature of critical illness and is recognized as an independent predictor of disease progression and mortality [28,29,30,31]. Our study contributes to this body of knowledge by providing novel evidence that ovarian hormone depletion affects not only cerebral parenchymal vessels but also dural microvascular compartments.

In the present study, we demonstrate that ovariectomy induces marked and compartment-specific remodeling of the CDM microvascular networks, with the arterial compartment being more profoundly affected than the venular counterpart. Quantitative morphometric analyses revealed significant reductions in both total and segmental vessel lengths, accompanied by increased vessel tortuosity in OVX group of animals. Notably, the post-OVX decrease in total vessel length was confined to the arterial compartment (Figure 3), indicating a selective vulnerability of arteriolar structures to estrogen depletion. In contrast, the venular compartment exhibited a relative resistance to OVX-induced changes, with only minimal or no detectable alterations in venular morphology. These findings suggest that ovarian hormone deficiency selectively compromises arterial microvascular integrity, which may have broader implications for tissue perfusion and vascular function in postmenopausal and post OVX states.

The reduction in arteriolar length, coupled with increased tortuosity, suggests active structural remodeling rather than passive degeneration. These changes are likely driven by the loss of well-established vasoprotective effects of estrogens, including their role in promoting endothelial integrity, inhibiting vascular inflammation, and modulating smooth muscle tone and proliferation [4,32,33,34]. This remodeling involves changes in the vessel wall structure and composition, leading to a shorter vessel but with abnormal twisting and turns. Furthermore, although not investigated in this study, factors like changes in elastin and collagen composition (ECM modification) or inflammatory processes could also play a role. There is the possibility as well that, due to the lack of negative feedback in the hypothalamic–pituitary–ovarian axis, FSH/LH levels may rise, potentially affecting microvascular changes. However, this possibility was not investigated in the current study.

The absence of a significant change in blood vessel surface area, despite the reduction in microvessel length, suggests the presence of compensatory dilation or vascular remodeling. This may serve to preserve perfusion and maintain mechanical homeostasis within the dura mater. However, direct quantification of vessel diameters was not performed in this study. As such, the interpretation of structural compensation remains speculative and highlights the need for future investigations employing diameter-based metrics. Also, even though most of the changes in blood microvasculature revealed in this study were associated with arteriolar networks, our investigation focused on the local consequences of OVX on the dura microvasculature. Therefore, we cannot extrapolate these results to the systemic microcirculation or speculate on their potential effects on total peripheral resistance (TPR) and associated effects on blood pressure. While there is a considerable body of research on OVX impacting blood pressure in rodents (primarily in rats), the reported results vary depending on factors such as age, time post OVX, experimental duration, and other conditions. However, the changes in dura mater microvasculature detected in this study, such as increased vessel tortuosity, while unlikely to affect TPR, may lead to disturbed blood flow patterns. These alterations can affect local wall shear stress and potentially contribute to conditions such as the development of dural arteriovenous fistulae.

In contrast to the relatively uniform responses of the arterial component of microvascular networks, venular vessels exhibited only minimal remodeling, suggesting that regulatory influence of ovarian hormones may be more pronounced in high-resistance vascular beds. This distinction may stem from intrinsic differences in endothelial phenotypes or from differential expression of estrogen receptors between arterial and venous vessels in the dura mater [4,8,35,36,37].

In parallel, our observations of vascular alterations, including increased α-SMA expression, are consistent with pathological vascular remodeling. Recent evidence indicates that α-SMA expression increases in brain pericytes following vascular lumen obstruction in both young and old mice; however, aged animals display a diminished capacity to upregulate this contractile protein sufficiently, which may limit their ability to maintain vascular tone and integrity under stress conditions [37]. Elevated α-SMA in vascular mural cells, particularly pericytes, is increasingly recognized as a hallmark of vascular dysfunction, and has been implicated in diseases such as diabetic retinopathy and Alzheimer’s disease [38,39]. Excessive α-SMA expression in these cells can contribute to vessel constriction, reduced cerebral blood flow, and capillary rarefaction [40].

Moreover, vessel shortening or remodeling, induced by altered hemodynamics or matrix composition, can further exacerbate vascular pathology. Such mechanical stressors have been shown to drive increased vascular smooth muscle cell (VSMC) coverage, neointima formation, and shifts in VSMC phenotype toward a proliferative or synthetic state [41,42]. These changes are associated with apoptosis, ECM remodeling, and loss of contractile markers. In the context of OVX, estrogen loss may potentiate these maladaptive vascular responses by disrupting endothelial–mural cell interactions and altering TGF-β signaling. TGF-β is a key driver of myofibroblast differentiation and extracellular matrix production, linking ovarian hormones deprivation to connective tissue remodeling in the dura mater [43,44]. These changes likely contribute to a stiffer and more fibrotic meningeal environment following OVX.

In the coronal suture region, a consistent anatomical landmark where we previously identified PDPL-positive lymphatic networks [20], we observed significant lymphatic vascular remodeling with the fraction of PDPL-positive lymphatic vessels increased in OVX animals. A simultaneous significant increase in the number of PDPL-positive lymphatic endothelial progenitor cells within the same CS regions further supports an active lymphangiogenic process [24] following ovarian hormone depletion. Given growing evidence that lymphangiogenesis contributes to fibrosis in various organs [45], our observations may reflect the early onset of profibrotic changes in the CDM as soon as two weeks post-ovariectomy. Considering the role of meningeal lymphatics in cerebrospinal fluid drainage and immune cell trafficking, such alterations may influence CNS homeostasis and neuroimmune interactions following OVX [17,18].

Our findings extend current understanding of the consequences of sex hormones perturbations by demonstrating that ovariectomy not only induces vascular changes but also prompts cranial dura mater stromal remodeling. Specifically, we observed a significant increase in α-SMA expression in the stromal compartment, indicative of myofibroblast activation and initiation of fibrotic pathways [46,47]. This was accompanied by elevated levels of the TGF-β, a central mediator of fibrosis, as shown by Western blot analysis (Figure 8) [48]. TGF-β is a well-characterized driver of fibroblast activation and ECM deposition, both of which contribute to connective tissue stiffening and structural remodeling [43,44]. These data suggest that ovarian hormones play protective role in maintaining meningeal tissue homeostasis, and that their depletion rapidly initiates a fibrotic program within the dura mater. This in turn supports the novel concept that the meninges are a hormonally sensitive tissue compartment, with potential relevance for neuroinflammatory or fibrotic conditions associated with menopause and sex hormone decline.

Taken together, our findings provide new insights into how the loss of ovarian hormone production selectively alters meningeal vascular architecture. The predominance of arterial and lymphatic remodeling underscores the vulnerability of specific vascular compartments to hormonal regulation. These structural changes may have functional implications for cranial vascular tone, neuroimmune signaling, and disease susceptibility in postmenopausal states, such as migraine, neuroinflammation, and cognitive decline. Our data also reveal a convergence of stromal fibrosis and vascular remodeling in the dura mater following the cessation of ovarian hormone production. These findings support a model in which hormonal decline precipitates both connective tissue remodeling and vascular dysregulation—two processes that may act synergistically to impair meningeal function. Future studies should investigate the temporal dynamics of these changes and determine whether they are reversible through hormone replacement therapy or pharmacologic inhibition of fibrotic pathways. Collectively, our findings suggest that the CDM is a hormonally responsive structure and may represent a critical site of vascular vulnerability in states of estrogen deficiency. Given the close interplay between blood and lymphatic networks within the dura, further research is warranted to elucidate how estrogen modulates these systems and to explore the broader implications for cerebrovascular health and neuroinflammation in estrogen-depleted conditions.

## 5. Conclusions

Our findings demonstrate that the loss of ovarian hormone production induces selective remodeling of meningeal vascular architecture, with pronounced changes in arterial and lymphatic compartments. These alterations are accompanied by stromal fibrosis, suggesting a coordinated response involving both connective tissue and vascular systems. Together, these changes may compromise meningeal function and contribute to increased susceptibility to postmenopausal conditions such as migraine, neuroinflammation, and cognitive decline. The dura mater, and specifically the CDM, emerges as a hormonally responsive structure and a potential site of vascular vulnerability in estrogen-deficient states. Future studies are warranted to define the reversibility and functional consequences of these changes, and to explore therapeutic strategies targeting hormone-responsive and fibrotic pathways in the meninges.

## Data Availability

Data is contained within the article and Supportive Information.

## Supplementary Materials

The following supporting information can be downloaded at https://www.mdpi.com/article/10.3390/cells14211647/s1, Table S1: FILE_Blood vessel morphometry_IF_vs_OVX; Table S2: FILE_Vessel_tortuosity_IF_vs_OVX; Table S3: FILE_OVX_vs_sham_Coronal_Suture_PDPL+ vessel_area & cell count; Table S4: FILE_aSMA coverage_OVX_vs_Sham; Table S5: FILE_TGF-beta densitometry_OVX_vs_sham.
